# Research Progress of the Preparation of Cellulose Ethers and Their Applications: A Short Review

**DOI:** 10.3390/molecules30071610

**Published:** 2025-04-04

**Authors:** Meng He, Yanmei Lin, Yujia Huang, Yunhui Fang, Xiaopeng Xiong

**Affiliations:** 1 KZJ New Materials Group Co., Ltd., Xiamen 361199, China; hemeng666@ycit.edu.cn (M.H.); 15759281170@163.com (Y.L.); 2 Key Laboratory for Advanced Technology in Environmental Protection of Jiangsu Province, School of Materials Science and Engineering, Yancheng Institute of Technology, Yancheng 224051, China; huang13913906481@163.com; 3 Department of Materials Science and Engineering, College of Materials, Xiamen University, Xiamen 361005, China

**Keywords:** cellulose ethers, concrete and mortars, etherification processes, modification, applications

## Abstract

Cellulose ethers (CEs), synthesized through the etherification of cellulose, have emerged as indispensable “green additives” in our modern industries, earning the moniker of industrial “monosodium glutamate” due to their unparalleled multifunctionality. Unlike traditional petroleum-based modifiers, CEs offer a unique combination of renewability, low toxicity, and tunable properties (e.g., water retention, thickening, and stimuli-responsiveness), making them pivotal for advancing sustainable construction practices. This review presents an overview of the preparation methods of various CEs and the applications of CEs especially in concrete and mortars as well as corresponding mechanisms. We systematically analyze the preparation methodologies (homogeneous vs. heterogeneous processes) and highlight the effect of molecular determinants (degree of substitution, molecular weight, functional groups) on the performances of CEs. CEs can enhance the workability and other properties of concrete and mortars primarily by acting as water-retaining and thickening agents to mitigate rapid water loss, improve hydration efficiency and cohesion. The effects of CEs on the delay of hydration and microstructure of concrete and mortars are also analyzed and highlighted. Beyond construction, we reviewed the current and emerging CE applications in biomedicine, tissue-engineering, petroleum industry and food engineering, highlighting their cross-disciplinary potential. This review provides some insights into the structure–property–application relationships of CEs and their brief historical developments, offering guidance for optimizing their utilizations especially in sustainable construction practices.

## 1. Introduction

As one of the largest energy sources on the earth, biomass exhibits attracting renewability and wide-availability, which can satisfy the requirements of constructing a low energy, efficient and environmental-friendly energy system and is coded as “cornerstone of the global bioeconomy” [1]. Undoubtedly, the utilization of biomass-derived resources has garnered increasing interest as a promising strategy to facilitate the attainment of “carbon emission peak” and “carbon neutrality objectives” [2]. With an annual production exceeding 75 billion tons, cellulose is the most abundant biopolymer in the world, offering inherent advantages of wide-availability, biocompatibility, biodegradability, and chemical versatility [3]. Moreover, abundant cellulose-based materials with various materials forms, including fibers, hydrogels, beads, aerogels, sponges, and microspheres, have been be prepared facilely via direct extraction or dissolution [3], regeneration process and of cellulose raw materials as well as fermentation methods for bacterial cellulose [4], which have been used in various fields including textile, biomedical, packaging, water treatment, and optical devices, among others [5].

However, cellulose is infusible and difficult to dissolve in common solvents due to the intramolecular and intermolecular hydrogen bonds that have greatly restricted its applications. It is noted that the preparation of soluble cellulose derivatives in water or common solvents, such as cellulose esters or cellulose ethers (CEs), can solve this problem effectively on the basis of the special structure of cellulose and chemical reactivity, which could endow cellulose with other amazing properties, including high water retention and antibacterial properties as well as stimulus responsiveness [6,7]. Historically, cellulose’s reactivity was used through chemical derivatization as early as 1846, when Christian Friedrich Schönbein pioneered nitrocellulose (“gun cotton”, a typical cellulose ester), revolutionizing explosives and the film industry through esterification [8]. Compared with cellulose esters, CEs usually exhibited better water solubility, temperature adaptability, biodegradability and water retention (thickening), which are synthesized and more eco-friendly [9,10]. The industrialization of cellulose esters in the mid-19th century provided foundational technologies (e.g., cellulose purification, reaction optimization) for the later development of CEs in the early 20th century. CEs are synthesized via etherification reactions that functionalize hydroxyl groups on the anhydroglucose unit (AGU) (the fundamental monomer of cellulose) with targeted pendant groups [3]. There are many types of CEs, and some common CEs can be classified according to differences in their substituents, ionization, and solubility (Table 1) [6,11].

According to the types of substituents, CEs are categorized into single ethers and mixed ethers [11]. CEs can also be categorized into water-soluble (e.g., MC, CMC, HEC, and HPC) and insoluble CEs (e.g., EC, CEC) according to their solubility. Water-soluble CEs can be further divided into ionic (e.g., CMC), non-ionic CEs (e.g., MC, EC, HEC, and HPC), and mixed ionic CEs (e.g., CMHEC, HPCMC, and HECMC) according to the ionization properties of substituents. CEs demonstrate tunable physicochemical properties through precise control of degree of substitution (DS) and molecular weight (M_w_). Critical parameters of CEs are M_w_/viscosity, DS, and molar degree of substitution (MS) with hydroxyalkyl groups per AGU [12].

Many different methods have been developed and reported for the fabrication of different CEs, and part of the methods have realized industrialized production since the 1920s, whose products have been utilized widely in our daily life including food, pharmaceutical, personal care products, oil field chemicals, construction materials and specialty chemicals (adhesives, textile sizing agents, paper coatings), etc., [6,13].

As a class of versatile biopolymers, CEs have garnered recognition as “industrial monosodium glutamate” owing to their indispensable multifunctionality across diverse sectors. The synthetic routes of different CEs are represented in this review together with their characteristics. This review mainly focuses on their effects and applications in cement-related materials like concrete and mortars. Moreover, their advantages and mechanisms, especially in cement-related materials, are also highlighted. Finally, the prospect and limitation of CEs are described in brief, which could possibly give some theoretical guidance on the fabrication of novel CEs and further broadening the application of CEs.

## 2. Fabrication Process of Different CEs

### 2.1. Heterogeneous Production of CEs

The industrial synthesis of CEs has historically relied on heterogeneous aqueous-phase systems, necessitated by cellulose’s inherent insolubility in conventional solvents [14]. This conventional protocol involves sequential steps: (1) alkali activation using concentrated NaOH under mechanical agitation, (2) removal of excess alkali, (3) etherification with alkyl halides or epoxides, and (4) purification through neutralization, washing, and drying. Despite its early industrial adoption since the 1920s, this aqueous-phase method suffers from critical limitations, including low etherifying agent utilization (60–70%) and uncontrolled side reactions (e.g., hydrolysis of etherifying reagents), resulting in inhomogeneous substitution patterns and residual byproducts [15,16]. To address these challenges, organic solvent-mediated heterogeneous systems have emerged as the dominant industrial approach for commercial CE production (e.g., MC, CMC, HEC and HPC) [17]. The key technical advancements include three aspects: (1) Solvent selection: Employment of inert dispersants (ethanol, isopropanol, tert-butanol) that enhance cellulose accessibility while resisting etherification side reactions; (2) process optimization: controlled NaOH incorporation in solvent-cellulose suspensions and gradual reagent addition under optimized mass/heat transfer conditions; and (3) recycling infrastructure: closed-loop solvent recovery systems mitigating operational costs [14]. The utilization rate of etherifying agents is increased by 10–20% for this method compared with the above heterogeneous aqueous method due to the enhanced reagent diffusion kinetics within swollen cellulose microfibrils, suppressed hydrolysis pathways due to reduced water activity, and improved thermal homogeneity during exothermic etherification [14]. Moreover, the reaction stability and uniformity are high, and the product performance is also significantly improved. However, this method is relatively costly (about 25–40% higher than conventional aqueous processes), which is primarily driven by solvent procurement and recycling expenses [16].

**Figure 1:**
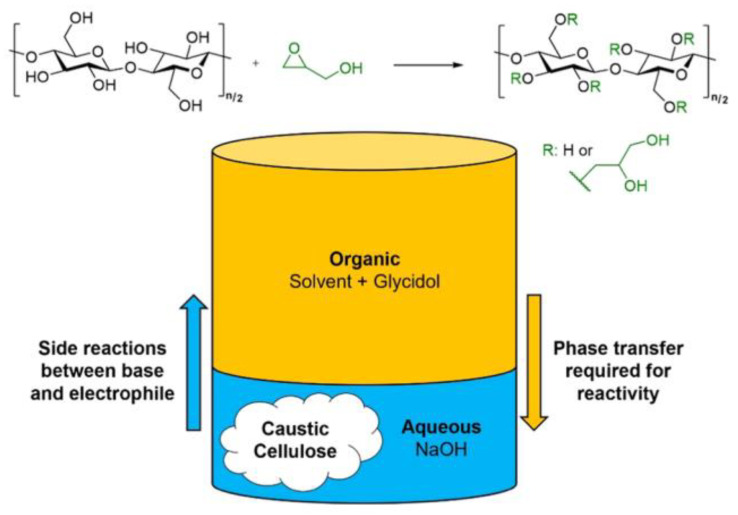
(**Top**): General scheme to synthesis DHPC. (**Bottom**): Illustration showing the three-phase slurry and the consequences of reagent transfer between phases. Reprinted with permission from ref. [17]. Copyright (2024), ACS Applied Polymer Materials.

Beyond industrial-scale production, heterogeneous synthesis strategies have demonstrated significant potential in laboratory-scale development of novel CEs. A notable advancement was reported by Kim et al. [17], who successfully synthesized 2,3-dihydroxypropylcellulose (DHPC) through a heterogeneous slurry-phase system employing cellulose and glycidol (Figure 1). Cellulose was activated in an organic solvent spiked with NaOH aqueous solution, followed by the controllable etherification reaction with glycidol. They pointed out desired DHPC with high solubility and high viscosity and could be obtained with high yields by controlling MS of glycidol units per AGU in the range of 1.0–2.0. The methods and trends highlighted in this work can be generalized to other cellulose derivatives and provide a starting point for future research and development of cellulosic materials with greener syntheses, tunable properties, and accurate characterization methods for use in a variety of aqueous applications.

**Figure 2:**
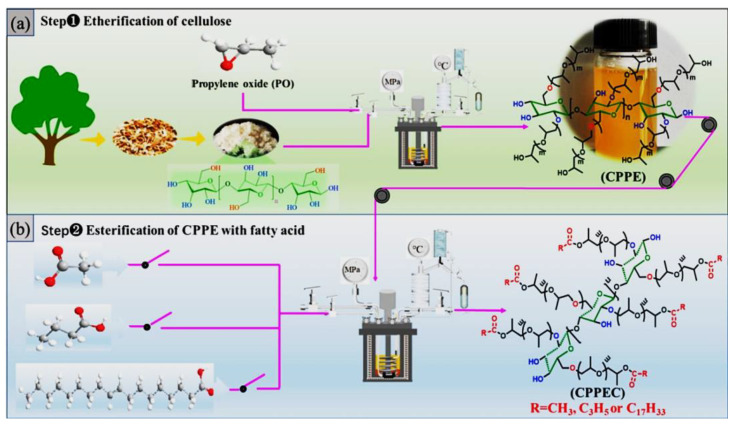
Synthesis of cellulose-based polypropoxy ether carboxylates (CPPEC). Reprinted with permission from ref. [18]. Copyright (2023), International Journal of Biological Macromolecules.

Cellulose-based polypropoxy ether carboxylates (CPPEC) with high blending compatibility were firstly fabricated by cellulose propoxylation and the following esterification with acetic acid, butyric acid, and oleic acid, respectively (Figure 2) [18]. Propylene oxide was used to modify alkali cellulose to prepare cellulose-based polypropoxy ether (CPPE) by a direct etherification process. The resultant CPPEC was used to plasticize PLA, which could intersperse within the PLA intermolecular spaces. Moreover, the synergistic effect of propyl and methyl groups in CPPEC with PLA matrix could obviously improve the mechanical properties and resistance of low-temperature or migration for PLA.

In the heterogeneous etherification of cellulose, the solid cellulose is usually suspended only in the liquid reaction media, so the inhomogeneity of the structure cellulose raw materials prevents the homogeneous reaction, which is limited to the surface and the amorphous region, resulting in the limitations of low DS, low purity, and uniformity of the products [16]. Thus, the product purity and uniformity became the main problem of the heterogeneous method.

### 2.2. Homogeneous Preparation Methods of CEs

To overcome the inherent limitations of heterogeneous cellulose etherification, homogeneous reaction systems have emerged as a transformative approach enabled by advanced cellulose-dissolving solvents. These systems achieve molecular-level accessibility of cellulose hydroxyl groups, permitting regioselective and uniform substitution patterns, which have become more and more popular due to the development of various novel cellulose solvents [19], including NaOH/urea, LiCl/DMAc, tetra-n-butylphosphonium hydroxide ([P_4,4,4,4_]OH) and DMSO/tetrabutylammonium fluoride (TBAF), among others, which could effectively control the regular introduction of substituent groups to the cellulose backbone [15,20,21,22,23].

Homogeneous etherification of cellulose can control the physical and chemical properties of the resultant CE products better than the heterogeneous reactions and is beneficial to improve the uniformity of product properties for the broadening of their application range [24]. Moreover, the cellulose homogeneous etherification reaction does not need to consider the problem of the reagent’s penetration rate into cellulose due to the dissolution in solvents, which is conducive to improve the reaction rate. The homogeneous reaction rate of cellulose is usually an order of magnitude higher than that of the heterogeneous reaction rate. Therefore, the homogeneous etherification of cellulose is of great significance as long as the reaction conditions and chemical reagents are selected appropriately, and the reaction process can be effectively controlled to obtain the expected CEs products, which have become more and more popular in recent years [20].

It is noted that various etherification methods from homogenous systems have been developed to reduce cost and increase reaction efficiency and homogeneity. The major fabrication methods and corresponding mechanisms for CEs are presented and summarized here. Zhang’s group has developed a series of non-toxic and pollution-free cellulose solvent systems, namely alkali/urea aqueous solution systems, which can be used as stable reaction media to uniformly synthesize various CEs, such as MC, HEC, HPC, CEC, and CMC, under mild conditions [24,25,26,27,28,29,30]. Due to the presence of alkali in the solvent system, it is not necessary to add additional bases as catalysts during the reaction.

**Figure 3:**
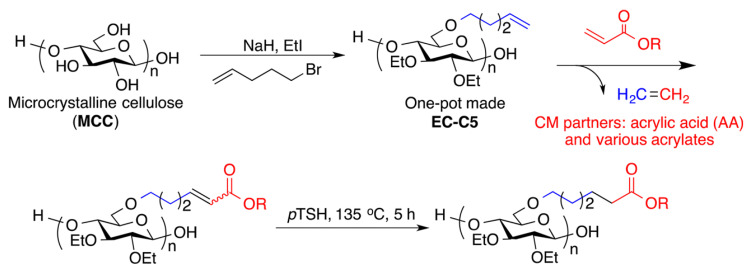
Synthesis of EC2.30C5, CM, and hydrogenation of CM Products. Reprinted with permission from ref. [31]. Copyright (2016), Biomacromolecules.

Olefin cross-metathesis (CM) is an important approach to graft various functional groups onto CEs, which could be used to fabricate a series of functional derivatives with high efficiency at a relatively mild condition [31]. Metathesis is used to modify EC for the preparation of useful derivatives with improved solubility and bioavailability of drugs, but it is usually restricted by low hydroxy DS for commercial EC derivatives. Edgar’s group have synthesized EC derivatives by a homogeneous one-pot process directly from LiCl/DMAc dissolved cellulose (Figure 3) [31], which exhibit controllable substituent DS as the ω-unsaturated alkyl groups and required CM substitution and hydrophilicity for the preparation of more hydrophilic derivatives, showing promising potential for solid dispersion etc.

**Figure 4:**
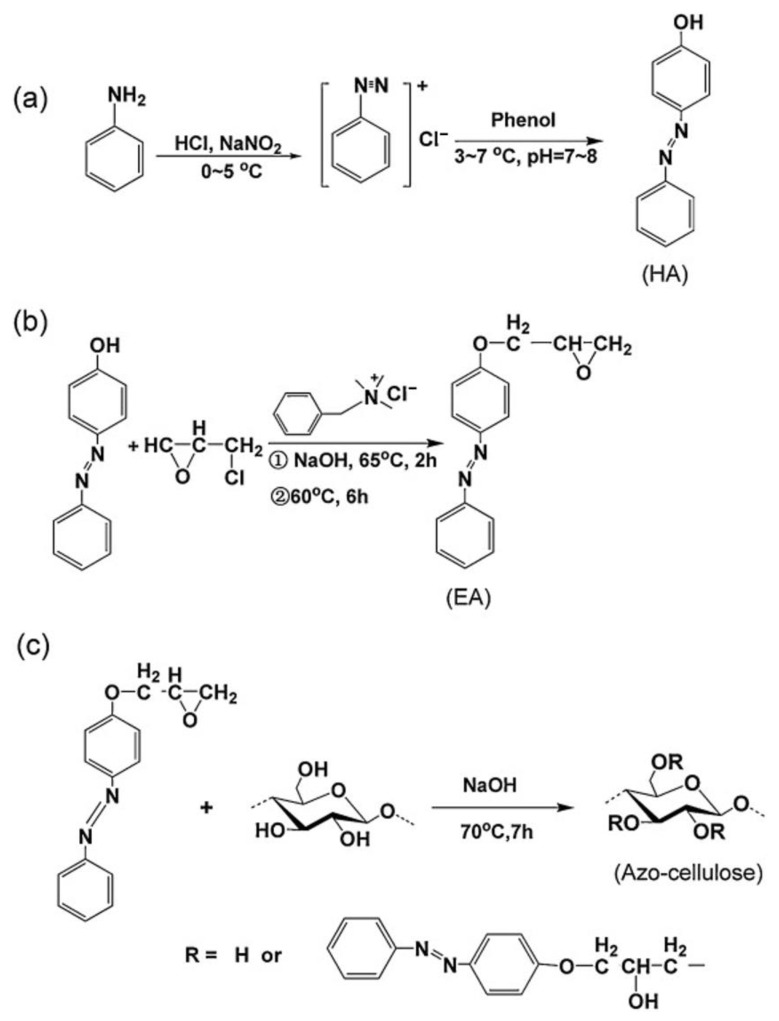
Synthetic route of (**a**) HA, (**b**) EA, and (**c**) Azo–cellulose. Reprinted with permission from ref. [19]. Copyright (2014), Carbohydrate Polymers.

Through homogeneous etherification of cellulose with 2,3-epoxypropoxy-azobenzene (EA) in DMAc/LiCl solvent system, Liu’s group successfully synthesized photoresponsive 3-azobenzene-functionalized CEs (Azo–cellulose) (Figure 4) [19]. The resulting CEs demonstrated reversible photoisomerization behavior under alternating 365 nm UV and visible light irradiation, with remarkable trans-cis switching efficiency. This photochromic functionality was attributed to the precisely engineered azobenzene moieties grafted onto the cellulose backbone.

**Figure 5:**
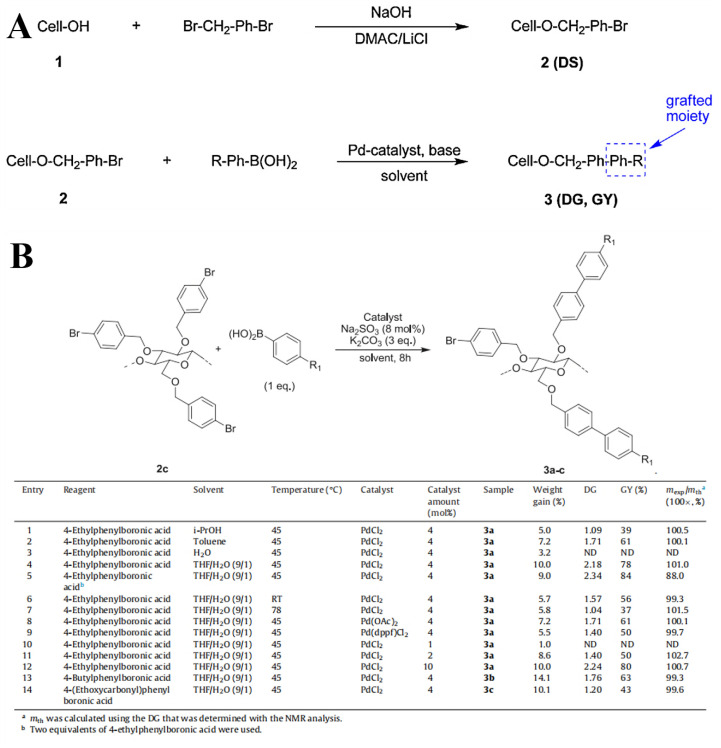
Definition of the weight gain, DS, DG, GY, M_(3)_ and m_th_ (**A**), scheme and results for the fabrication of various CEs (**B**). Reprinted with permission from ref. [33]. Copyright (2015), Carbohydrate Polymers.

The Suzuki–Miyaura cross-coupling reaction, a transformative palladium-catalyzed carbon–carbon bond-forming methodology first developed by Akira Suzuki and Norio Miyaura in 1979, has emerged as a fundamental tool in modern synthetic chemistry owing to its remarkable versatility and compatibility with mild reaction conditions [32]. Leveraging this groundbreaking platform, Goncalves and colleagues demonstrated an innovative adaptation of the Suzuki–Miyaura protocol for cellulose modification, establishing an efficient pathway to synthesize a novel class of CEs with precisely engineered functionalities (Figure 5) [33]. Their methodology enabled the controlled incorporation of diverse alkyl chains and functional groups onto the cellulose backbone and maintained structural integrity at the same time. Notably, this advancement addressed the long-standing challenge of grafting sensitive functional moieties to biopolymer scaffolds under exceptionally mild conditions, thereby expanding the potential for designing advanced cellulose-based materials with tailored physicochemical properties.

Benzyl cellulose has demonstrated significant potential as a hemodialysis membrane material, representing a crucial advancement in biomedical applications [34]. Conventional heterogeneous synthesis methods employing concentrated NaOH aqueous solutions and benzyl chloride by non-uniform product distribution and DS values have been limited, primarily due to the layer-by-layer benzylation mechanism [34,35]. To overcome this issue, Rohleder et al. utilized a DMSO/tetrabutylammonium fluoride (TBAF) solvent system to improve reaction homogeneity significantly [23]. Moreover, Sun’s research group achieved a breakthrough through homogeneous etherification in cold cellulose/NaOH/urea aqueous solutions, successfully synthesizing benzyl cellulose with tunable DS values ranging from 0.29 to 0.54 under mild reaction conditions [29]. This aqueous-phase methodology not only enhances substitution uniformity but also aligns with green chemistry principles by eliminating organic solvents.

**Figure 6:**
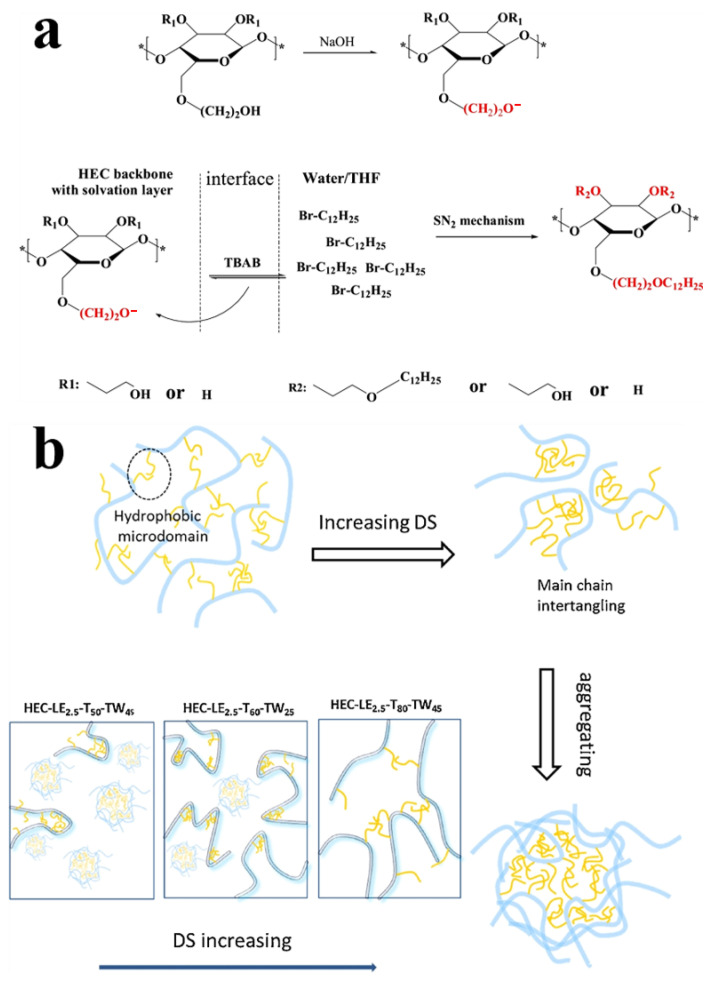
Reaction mechanism of HECLE (**a**) and the influence of DS on the solution structure of HECLE (**b**), * represent chain end groups or unspecified terminal groups. Reprinted with permission from ref. [36]. Copyright (2019), Colloids and Surfaces A: Physicochemical and Engineering Aspects.

The development of amphiphilic copolymers through strategic modification of HEC has emerged as an effective approach to combine hydrophilic and hydrophobic functionalities. Ran’s research team successfully demonstrated this concept by synthesizing hydroxyethyl cellulose lauryl ether (HECLE) derivatives through phase-transfer catalyzed etherification [36]. Their methodology employed 1-bromododecane as the alkylating agent in a binary solvent system of water and tetrahydrofuran, with tetrabutylammonium bromide (TBAB) serving as the phase-transfer catalyst (Figure 6). This approach yielded HECLE polymers with precisely controlled DS ranging from 0.21 to 1.95, enabling systematic investigation of structure–property relationships. The synthesized amphiphilic derivatives exhibited characteristic self-assembly behavior in aqueous media, forming nanoaggregates through hydrophobic interactions. Notably, modified HECLE derivatives demonstrated enhanced rheological properties compared to native HEC and improved thickening efficiency. These tunable physicochemical characteristics position HECLE as a promising material for multifunctional applications, including protective coatings, encapsulation systems for hydrophobic actives, and rheology modifiers in industrial formulations.

**Figure 7:**
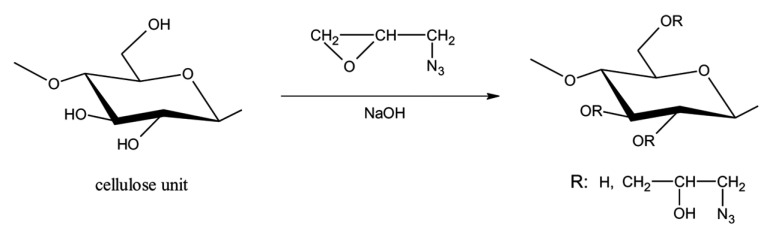
Scheme for the preparation of 1-azido-2-hydroxypropyl cellulose ether. Reprinted with permission from ref. [37]. Copyright (2011), Journal of Energetic Materials.

Yang and co-workers reported the innovative preparation of 1-azido-2-hydroxypropyl cellulose ether through a sequential ring-opening and etherification strategy, wherein cellulose was reacted with glycidyl azide in the presence of diazoimide (Figure 7) [37]. The epoxy group of glycidyl azide underwent ring-opening etherification with cellulose hydroxyl groups, enabling the covalent attachment of azide-functionalized moieties to the polysaccharide backbone. This methodology not only introduced nitrogen-rich azide groups with high energetic density but also preserved the structural integrity of cellulose. Notably, the resulting cellulose derivative exhibited tunable physicochemical properties and remarkable energetic characteristics, making it as a promising candidate for advanced energetic materials. The work demonstrated a scalable approach to functionalize biopolymers with reactive groups while addressing challenges in balancing stability and energy output for practical applications.

The strategic grafting of azobenzene moieties onto biopolymers offers a promising route for developing biodegradable photochromic materials. In a notable demonstration, two photoresponsive azobenzene-functionalized hydroxypropyl cellulose derivatives (azo-HPCs) were homogeneously fabricated via the etherification of HPC with either 4-(2′,3′-epoxypropoxy)azobenzene or bromoethoxyazobenzene in DMAc [38]. These derivatives exhibited reversible trans–cis–trans photoisomerization behavior, showing light-responsive characteristics. Furthermore, the azobenzenoxy–ethoxy–HPC (azo–EHPC) derivative displayed thermotropic liquid crystalline properties, forming a distinct cholesteric texture at 165 °C. This work highlights the potential of CE-based materials for advanced applications in stimuli-responsive optics and adaptive biomaterials.

Homogeneous methods present a valuable approach for investigating the etherification conditions of unconventional CEs, enabling enhanced control over product uniformity and DS. However, these methods currently face significant limitations: they often demand stringent cellulose dissolution conditions, and most of them are relatively costly reagents [17]. Additionally, scalability is hindered by challenges in processing high-viscosity cellulose solutions at elevated concentrations, which complicate large-scale manufacturing. Consequently, homogeneous systems face significant barriers to industrial adoption, unless they demonstrate unequivocal advantages over established heterogeneous methods. To advance their practicality, systematic optimization of reaction parameters, such as novel solvent systems, must be prioritized. Such efforts could pave the way for streamlined, low-cost processes to synthesize tailored CEs with precise functionality while mitigating current economic and technical constraints. The fabrication process can affect the critical parameters of CEs, such as M_w_, homogeneity, and DS, which further affect their applications.

## 3. Applications of Different CEs and Their Effects

As we mentioned above, CEs have various applications in different fields, which could also be further chemically modified to broaden their applications further [39,40,41]. In this review, we mainly focus on their applications in the building field, especially on concrete and mortars. Moreover, the corresponding mechanisms are also provided accordingly.

### 3.1. Applications and Effects of CEs on Concrete and Mortars

The rapid evolution of advanced construction technologies, such as 3D-printed concrete and self-leveling mortars that require precise workability control, has imposed increasingly stringent performance demands on modern cementitious materials [6]. To meet these challenges, tailored chemical admixtures are essential for optimizing both fresh-state properties (e.g., workability, adhesion) and hardened-state performance (e.g., strength, durability). CEs have emerged as multifunctional additives in concrete and mortar formulations, functioning as plasticizers, hydration delay modifiers, washout-resistant additives, and water retention promoters [42,43]. Their efficacy stems from their ability to modulate rheological behavior while maintaining colloidal stability in cement-water systems [44,45]. Critically, the functional performance of CEs is governed by formulation parameters (e.g., DS, M_w_), curing protocols, and environmental conditions. These factors intricately influence hydration kinetics, pore structure evolution, and long-term durability, necessitating precise dosage optimization for targeted applications. For instance, excessive CE content may delay hydration or induce undesirable porosity, whereas insufficient levels compromise workability and water retention [6]. Thus, a nuanced understanding of CE interactions with cement chemistry and processing variables remains pivotal for advancing next-generation sustainable construction materials.

#### 3.1.1. Improvement of Water Retention, Rheological Properties, Workability and Adhesion

Water retention refers to capacity that prevents fast water loss to the substrate by suction, which avoids bleeding or water loss and affects workability and adhesion between mortars (concrete) and substrates [44]. When contacting the supporting substrates, an insufficient hydration problem appeared for concrete and mortars due to the migration of water into the substrates during the curing stage of cement formation, resulting in a loss in the mechanical performances. Thus, pure concrete and mortars could not stratify the practical applications due to their poor water retention and rheological properties, which usually lead to bleeding, blockage, and even cracking [44].

**Figure 8:**
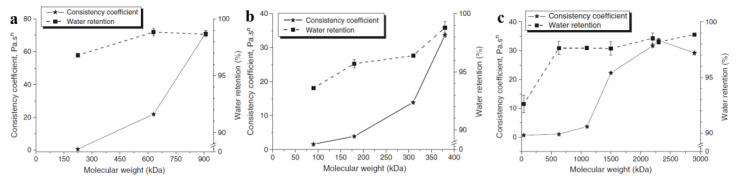
Effects of M_w_ of HPMC J (**a**), HEMC C (**b**), and HEC N (**c**) on the consistency coefficient and water retention of admixed mortars. Reprinted with permission from ref. [44]. Copyright (2011), Cement and Concrete Research.

Most CEs could effectively improve the water retention for proper hydration to fulfill the requirement of substrate adhesion through their hydrophilic groups, which is very important for their utilization as chemical admixtures in construction materials. Moreover, the effect of CEs on the freshly mixed mortars’ water retention is very important to guide its practical application. Patural et al. conducted a series of investigations to elucidate the role of CEs in cement-based system. They studied the influence of CE on the water mobility utilizing Nuclear Magnetic Resonance (NMR) at multiple scales [46]. In their findings, pulsed field gradient NMR analysis revealed that the addition of CE did not induce significant changes in the self-diffusion coefficient of water molecules, either in aqueous CE solutions or within hydrated cement pastes. This finding was further supported by interdiffusion imaging experiments showing comparable water mobility across the interface between fresh cement pastes, regardless of CE presence. Then, they systematically investigated the relationship between CE physicochemical characteristics (particularly M_w_ and DS) and macroscopic mortar performances, including water retention and rheological properties [44]. They highlighted the effect of M_w_ for CEs on mortar consistency; the mortar water’s retention capacity and consistency enhanced gradually with M_w_ on the whole, possibly due to enhanced steric stabilization effects, because larger CE molecules created more effective particle separation within the cement matrix through increased spatial hindrance (Figure 8a–c). HEMC with low M_w_ (90 kDa) had relatively low mortar water retention (below 94%) and HPMC with higher M_w_ (180–380 kDa) exhibited strong water retention capacities to the mortar (95.7–98.8%, respectively) (Figure 8b). Moreover, a retention capacity plateau (about 97.8%) appeared around 600 kDa, where M_w_ had little effect (Figure 8c). The results also indicated an obvious thickening effect with the 0.27 wt.% CE. CEs could be absorbed by Cement hydrates particles to form a coating on cement particles and reduce the contact of cement particles, acting as a dispersant. The coating and dispersant effects increased with the M_w_ for CEs.

**Figure 9:**
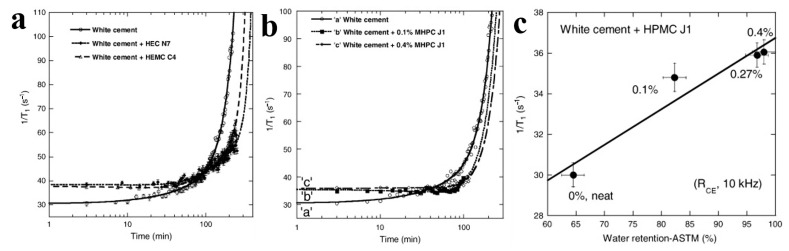
Water ^1^H spin–lattice relaxation rates of hydrated cement pastes as a function of hydrating time-effects of different CEs (**a**), the CE concentration of HPMC (**b**), and the correlation between the water retention evidenced from the macroscopic measurement and the NMRD method (**c**). Reprinted with permission from ref. [47]. Copyright (2012), Cement and Concrete Research.

Marliere et al. employed a multi-method experimental approach to unravel the mechanistic basis of water retention in a CE-modified porous media [45]. They combined standardized water retention assessments with specialized imbibition and filtration analyses using controlled model porous matrices and varied fluid compositions. CEs accumulated at constrictions and enlarged pore networks measuring 20–100 μm in diameter due to a critical pore-blocking phenomenon during water transport, whereas CEs mortar pore size was around 10 μm without CEs. The observed size disparity suggests potential CE-mediated modifications to both water retention behavior and filtration dynamics in cementitious systems, despite the fundamental differences between model and real-world porous architectures. Complementing their experimental findings, they developed a statistical filtration model capable of quantitatively reproducing key empirical patterns. This theoretical framework not only corroborated the experimental evidence for macromolecular jamming effects but also provided predictive insights into how CE characteristics might influence fluid transport regulation in heterogeneous porous environments. The combined experimental–modeling approach advanced understanding of CE functionality beyond empirical correlations, establishing a physical basis for optimizing water retention through molecular–scale interactions in construction materials. Nuclear magnetic resonance dispersion (NMRD) techniques have decoded CE’s water retention mechanisms at molecular interfaces. Patural elucidated the influence of CEs on water retention of freshly mixed white cement pastes by using NMRD [47]. They studied the influence of CE’ types and concentrations on the water transiently (Figure 9a,b). The macroscopic water retention of white cement containing 0.1% HPMC increased from 82.3% to 98.0% containing 0.4% HPMC, respectively. Moreover, the linear correlation (r^2^ = 0.93) of RCE and water retention of mortar admixed with different HPMC concentrations (0.1, 0.27 and 0.4%). The results confirmed NMRD’s effectiveness and the plateau R_CE_ value 0.4% HPMC enhances greatly with the CE concentration, and the NMRD results correlated with the macroscopic water retention on mortars (Figure 9c).

As a very important CE, HPMC could greatly improve the rheological properties of slag cement-based 3D printed concrete by using ≤0.2% content, and the introduction of 0.15% HPMC could endow stable printability [48]. HPMC was used to enhance the grouting effect of ultrafine cement and increase cement stability effectively [49]. Furthermore, HPMC could also greatly improve the air retention and workability of the iron tailings sand concrete paste and effectively optimize corresponding pore structure by using 0.6% content [50]. Experimental studies about calcium sulfoaluminate (CSA) cement system were performed, and the results demonstrated that both HEMC and HEC exhibit effective cement particle adsorption at concentrations <0.1% while maintaining good water retention performance [51]. Moreover, further study indicated that HEMC induced a 10% water retention enhancement in CSA mortars through pore structure reorganization [52]. These findings align with broader observations in cementitious material science, where controlled CE addition balances fluid transport regulation and matrix development [6,52]. Gu et al. investigated the effect of HPMC on the iron tailings-based autoclaved aerated concrete, and their results showed that HPMC molecular chains entangled with each other and formed a three-dimensional polymeric networks, significantly enhancing both viscosity and water retention capacity through physical entanglement mechanisms [50].

**Figure 10:**
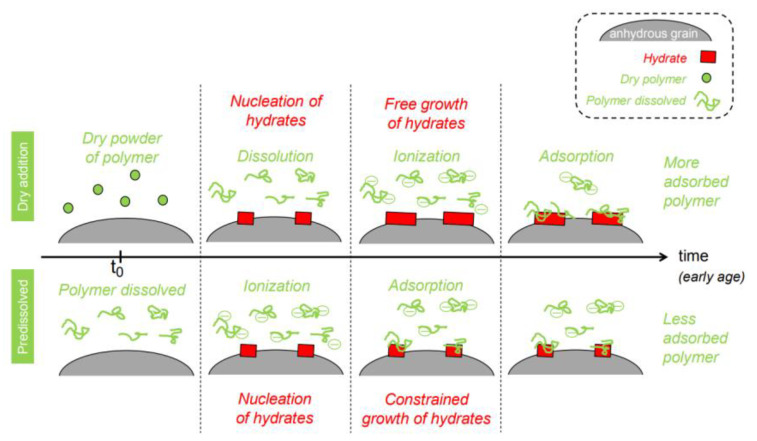
Scheme of the polysaccharide–cement interactions from different methods. Reprinted with permission from ref. [53]. Copyright (2015), Cement and Concrete Research.

Poinot et al. systematically investigated the influence of polysaccharides [e.g., HPMC and hydroxypropylguars (HCGs)] addition methods, either as dry powder or pre-dissolved in mixing water on the performance of fresh mortars [53]. Their findings revealed that pre-dissolving polysaccharides in water prior to mixing significantly enhanced water retention and rheological properties compared to dry powder addition. This method ensures immediate availability of dissolved polymer chains, which preferentially adsorb onto nascent hydration products during the initial mixing phase. Such adsorption inhibits the nucleation and growth of hydration phases, thereby reducing the reactive surface area available for further polymer binding (Figure 10). The adsorption on hydrated cement phases could be accelerated by pre-dissolved polysaccharides. This effect arises from rapid polymer adsorption onto early-stage hydrates, constraining their growth and limiting interfacial interactions. Conversely, dry powder addition resulted in slower polymer dissolution and adsorption, leading to higher overall polymer uptake but diminished functional efficiency. At equivalent dosages, dry-added mortars exhibited lower water retention, reduced consistency, and elevated yield stress values, alongside attenuated hydration retardation. The thickening behavior of polysaccharides was also highlighted: pre-dissolved polymers generated a shear-thinning rheological profile, improving workability under dynamic mixing conditions. Critically, the pivotal role of polymer conformation in governing the effectiveness of polysaccharides as multifunctional admixtures. These insights emphasize the necessity of optimizing additional protocols to balance hydration kinetics, rheological performance, and interfacial interactions in tailored mortar formulations. Apart from HPMC, other CEs (e.g., HEMC) could also improve the water retention by increasing the coefficient by at least 10% compared to mortars without CE [54].

It is noted that CEs can enhance the performance of concrete and mortars effectively, like working stability, even with a minimal amount, which can also improve the paste wrapping and cohesion [6]. The addition of CE could usually lead to air-entrainment, which could reduce the density to improve the workability and their utilization in walls or ceilings. CE incorporation diminishes water permeability by altering the material’s pore network architecture in model-hardened cement paste. This reduction stems primarily from air void formation induced by CE additives, which disrupts the continuity of capillary channels responsible for bulk water transport [55]. These findings established a physical basis for understanding how CE-mediated air entrapment modifies fluid transport dynamics in cement-based systems, independent of direct chemical interactions with the aqueous phase. In tile adhesives, renders, and self-leveling mortars, CEs improve adhesion to substrates by forming a cohesive matrix, which is critical for reducing cracks and enhancing long-term durability [56].

Zhang et al. have studied the influence of HPMC on mortar; they found that the addition of HPMC was a good thickener and could meet adhesion requirements, which had an obvious thixotropic effect on mortar [57]. Moreover, CEs improved the workability of fresh mortar and prolonged hydration, ensuring better adhesion and internal strength development [56].

Despite the utility of CEs in the improvement of above properties, their application faces critical challenges. First, achieving optimal functionality requires stringent dosage control, as even minor deviations can compromise water retention or workability. Second, the hygroscopic nature of CEs may exacerbate water sensitivity in cement blends, reducing their regulatory efficacy over time. Additionally, the inherent viscosity and poor dispersibility of CEs can hinder uniform distribution within the cement matrix, leading to particle aggregation and sedimentation. These issues collectively impair fresh-state properties, such as slump retention and pumpability, while potentially introducing inhomogeneities that affect hardened mechanical performance.

#### 3.1.2. Effects on the Delay of Hydration

Cement, as a critical component of concrete and mortars composed of clinker minerals, exhibits distinct hydration mechanisms and kinetic parameters during different stages of the hydration process, leading to diverse chemical and physical transformations. As we all know, CEs can be utilized viscosity-modifying agents in aqueous systems to retain water, which could slow water migration and affect the hydration kinetics, leading to longer induction and consolidation^3^ times of cementitious materials. Pourchez et al. demonstrated that CEs induce significant delays in cement hydration, ranging from 10 min to several hours, depending primarily on the molecular architecture and DS [58]. Further investigations utilizing isothermal calorimetry (IC) revealed that the molecular characteristics of CEs critically influence early-age hydration kinetics of Portland cement [59]. In their findings, the delay ability increased with the decrease in the M_w_ of CEs or the hydroxyethyl or methyl contents for different CEs. The delay mechanism was closely associated with the adsorption between CEs molecule and metal hydroxide on hydration products, and the adsorption ability of CEs increased with the decrease in the steric hindrance to hydroxyl groups for the CE chains. Similar results were also reported about the effects of DS or hydroxyethyl content on hydration delay [12,58]. Ciobanu et al. studied the effect of CEs (HEMC-related) on hydration kinetics of Portland cement using thermogravimetric analysis (TGA) [56]. Their results demonstrated that CE viscosity induced limited variations in Ca(OH)_2_ content, while the DS of CEs directly modulated the extent of cement hydration. CE additives significantly inhibit early-stage cement hydration (1–3 days), primarily through suppression of Ca(OH)_2_ formation, suggesting a structure–property relationship, in which polymer chain mobility and substitution density govern interfacial interactions with hydrating cement phases. The hydration delaying effect of CEs on polymer modified mortars was assessed as a function of the CEs’ incorporation timing and dosage rate. CEs’ delaying effect was closely related with its content in the mortar [12].

**Figure 11:**
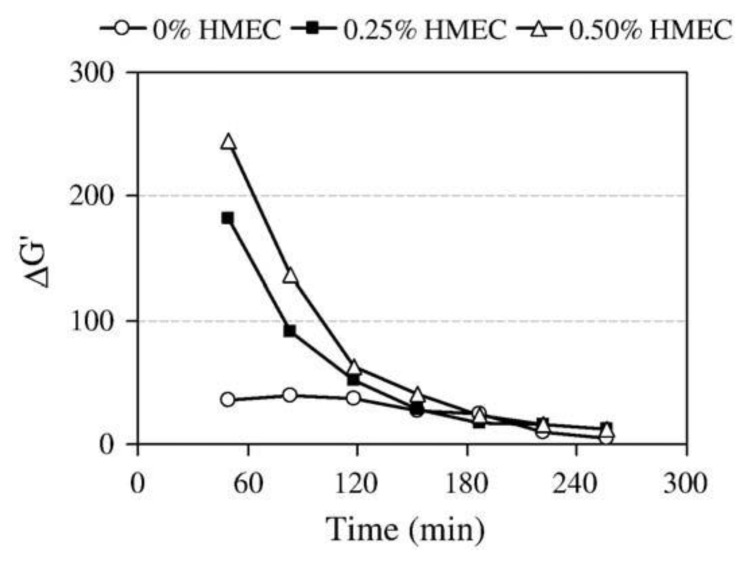
The ratio between the elastic components at the beginning and end of each oscillatory test. Reprinted with permission from ref. [60]. Copyright (2009), Cement and Concrete Research.

As for cement systems, CEs’ effect on the early hydration and rheology is very important for the selection and utilization of CEs. HMEC is extensively employed in self-compacting concrete and dry-mix mortars due to its exceptional viscosity-modifying performance. Pileggi’s group has studied the influence of HMEC on the consolidation process of cement pastes by evaluating rheological behavior via oscillatory rheometry and IC [60]. Their findings revealed that physicochemical interactions between HMEC and cement particles significantly amplify the complexity of the paste’s rheological behavior, mediated by alterations in particle agglomeration kinetics and hydration progression. During the initial hydration phase (within 2 h), HMEC exerts a dispersing effect through steric hindrance, effectively retarding the setting rate while enhancing system stability. This stabilizing effect intensifies with higher HMEC content, primarily attributed to steric repulsion caused by the adsorption of HMEC molecules on cement particles and calcium silicate hydrate (C-S-H) surfaces. The pastes with 0.25 and 0.50% HMEC had higher storage modulus (ΔG’) than the plain cement sample until 120 min, due to the increased elastic component of the polymer-containing sample, which became similar for pastes with or without HMEC after 120 min as a result of the hydration reactions and reagglomeration of particles (Figure 11). The rheological parameter analysis further demonstrated that HMEC incorporation reduces the minimum G’ and accelerates the modulus development rate, which are phenomena closely associated with the steric hindrance network formed by polymer chains and the hydrogelation process.

**Figure 12:**
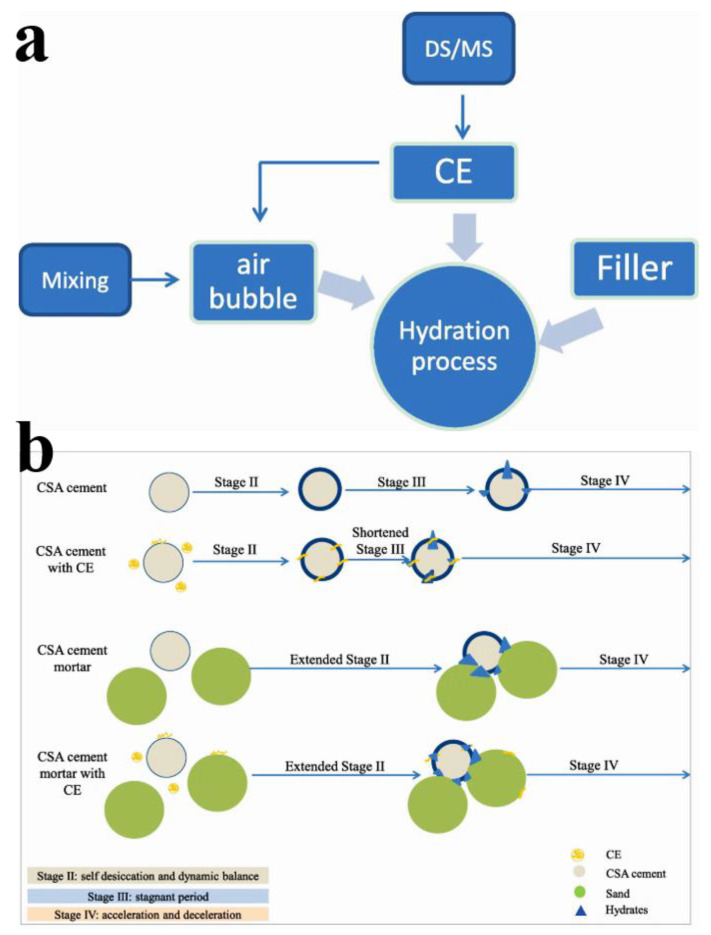
Probable factors affecting the hydration process of CSA cement/mortar (**a**) and the scheme of CSA cement/mortar hydration (**b**). Reprinted with permission from ref. [62]. Copyright (2022), Construction and Building Materials.

Zhang et al. systematically investigated the retarding effects of HEMC on the early hydration of calcium sulphoaluminate (CSA) cement using a multi-technique approach combining IC and TGA, etc. [61]. Their results demonstrated that HEMC postponed the occurrence time of two main heat flow peaks and decreased the early hydration degree. HEMC manifested significant retardation on the ettringite formation and amorphous aluminum hydroxide within 12 h of hydration but ensured higher ettringite content and lower contents of amorphous aluminum hydroxide and monosulfate in cement paste after 24 h. Moreover, HEMC’s effect on the early hydration evolution enhanced with the increase in its dosage. Recently, Wang’s research team conducted a comprehensive investigation into the effects of five CEs with distinct chemical structures or DS on the setting behavior and early hydration kinetics (<24 h) of CSA cement [62]. Through a multi-technique approach employing Vicat testing, ultrasonic pulse velocity monitoring, and IC, the study revealed significant variations in CE performance. Notably, HEMC and HPMC demonstrated a more pronounced prolongation of setting time compared to HEC. Furthermore, all CEs were found to accelerate the appearance of the second exothermal hydration peak in CSA cement, with HEC showing the most significant acceleration effect while highly substituted HEMC/HPMC varieties exhibited minimal impact. Interestingly, while CEs maintained similar hydration-influencing mechanisms in mortar systems as observed in cement paste, their overall effectiveness was substantially diminished. The researchers proposed a novel mechanism suggesting that CE films act as permeable media facilitating controlled water migration, thereby enhancing hydration reaction kinetics. This hypothesis was subsequently visualized through an innovative conceptual model (Figure 12) that elucidates the dynamic hydration process mediated by CEs. Apart from stage I, CSA cement/mortar hydration includes self-desiccation and dynamic balance (stage II), stagnant period (stage III), as well as acceleration and deceleration (stage IV).

Li et al. developed a five-phase hydration framework elucidating CE interactions in CSA cement matrices [62]. Their mechanistic analysis revealed CE’s dual functionality: significantly extending cement mortar setting durations while paradoxically accelerating the secondary hydration exotherm emergence, with these effects exhibiting strong dependence on CE chemical architecture and substitution patterns. Particularly, hydroxyethyl derivatives demonstrated greater temporal modulation capabilities compared to methyl-substituted counterparts, highlighting molecular structure-dependent interfacial behaviors. Emerging ultrasonic interrogation techniques have enabled real-time monitoring of CE-doped cement microstructural evolution [63]. Advanced non-destructive monitoring through ultrasonic propagation analysis quantified CE’s dosage-dependent retardation efficacy, demonstrating asymptotic behavior in setting time prolongation beyond critical additive concentrations. This methodology provided novel insights into the dynamic competition between CE’s water retention mechanisms and its interference with early hydration nucleation processes. The combined results establish a quantitative relationship between CE molecular parameters (DS, chain architecture) and their macroscopic performance in hydrating cement matrices.

**Figure 13:**
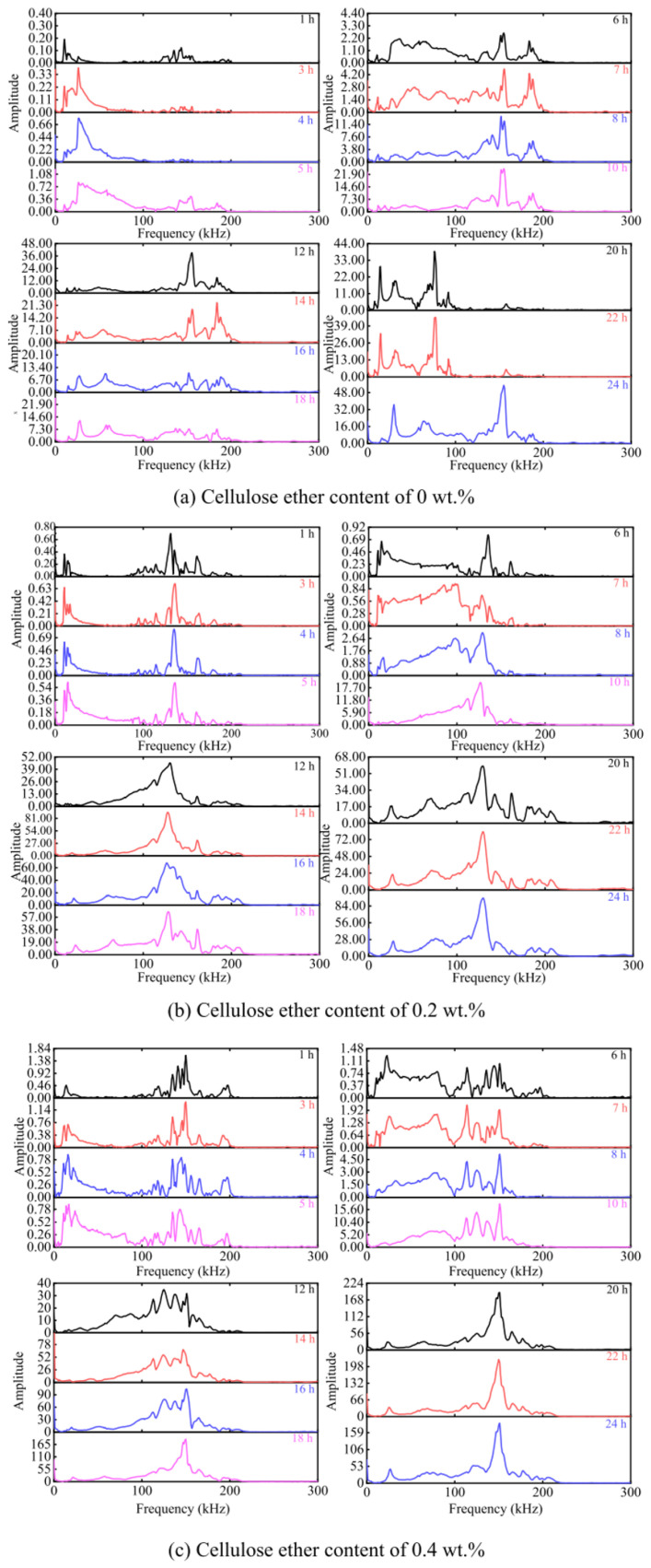
Frequency-domain spectra of cement pastes with different contents of cellulose ether at the early hydration period. (**a**) Cellulose ether content of 0 wt.%; (**b**) Cellulose ether content of 0.2 wt.%; (**c**) Cellulose ether content of 0.4 wt.%. Reprinted with permission from ref. [63]. Copyright (2024), Construction and Building Materials.

Hao et al. have studied the effects of different CEs on the hydration and hardening process of the cement paste using self-made piezoelectric ultrasonic transducers [63]. During the 6 h hydration process, the amplitude of high-frequency peaks exceeds that of low-frequency peaks, which is attributed to the water retention properties of CEs. Ultrasonic frequency-domain spectra, acquired via Fourier transform analysis (Figure 13), reveal that the dominant frequency peaks of ultrasonic waves propagating through hardened cement paste primarily concentrate within the 0–200 kHz range. Notably, after 20 h of hydration, the frequency-domain profiles of CE-doped cement pastes exhibit marked distinctions from those in Figure 13a, with distinct single dominant peaks emerging in the high-frequency regions of Figure 13b,c. This phenomenon may stem from the pore-filling action of CE gels within the cement matrix, which mitigates acoustic attenuation effects on high-frequency ultrasonic waves in CE-modified systems. Obviously, these findings collectively emphasize the structure-dependent role of CEs in modulating cement hydration processes.

**Figure 14:**
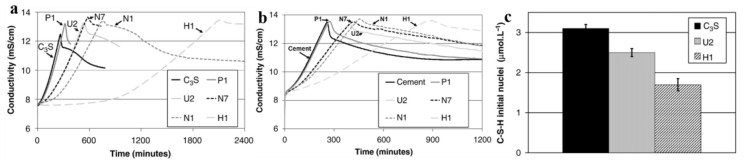
The impact of CE on the C_3_S hydration behavior (**a**), cement hydration behavior, (**b**) and the amount of initial nuclei of C-S-H precipitated after 30 min of C_3_S hydration (**c**). Reprinted with permission from ref. [65]. Copyright (2010), Cement and Concrete Research.

The study of the effect of CEs on the components of cement has a certain guiding significance for the study of their effect on the hydration delay of cement. The studies of the Pourchez and colleagues systematically explored the influence of CE chemical characteristics on tricalcium aluminate (C_3_A) (one component of cement) hydration mechanisms across progressively complex systems [64]. Adopting a systematic mineralogical approach, the researchers examined CE effects in isolated C_3_A phases before advancing to C_3_A–sulfate combinations, thereby establishing fundamental insights into CE-C_3_A interactions through controlled experimental analysis. Their methodology encompassed comprehensive evaluations of CE adsorption behaviors on key hydration products coupled with detailed monitoring of hydration progression in varied environments. Critical findings demonstrated CE’s rapid intervention in hydration processes, with experimental observations revealing significant retardation of C_3_A hydration kinetics within minutes of mixing initiation. This time-sensitive inhibitory effect was particularly pronounced in pure C_3_A systems, albeit modified by sulfate’s presence in the composite’s formulations. The study’s stratified experimental design effectively decoupled CE’s adsorption mechanisms from its broader chemical influences, providing critical baseline data for understanding polymer–cement interactions. Furthermore, Pourchez et al. investigated the impacts of various CEs on C_3_S dissolution, C-S-H nucleation-growth process, and cement hydration behavior by conductometry precipitation (Figure 14) [65]. During C_3_S hydration processes, where dissolution–precipitation kinetics compete, calcium-enriched CE additives exhibit inhibitory effects on C-S-H heterogeneous nucleation at C_3_S interfaces. The results indicated three characteristic impacts of CEs, namely (1) marked reduction in primary C-S-H nucleation density, (2) substantial alteration of C-S-H growth kinetics, and (3) phase-dependent retardation mechanisms varying with CE compositions.

#### 3.1.3. Effects on the Pore Structure and Mechanical Properties

The introduction of CEs can create obvious influence on the pore structure and mechanical properties, which usually associated the air voids within the cement hydration process for concrete and mortars [63]. The air-void architecture of CE-containing mortars exhibits distinct evolution patterns governed by additive type and dosage. Polished section analyses of tile-adhesive mortars revealed that increasing the HEMC/HPMC content from 0.3% to 0.8% (by dry mass) induced limited total porosity growth (<5% absolute increase) but significantly altered pore morphology [66]. Higher CE concentrations promoted air-void coalescence, reducing circularity indices and creating irregular, interconnected channels, which is a phenomenon attributed to polymer-mediated agglomeration during bubble stabilization. While ^1^H NMR relaxometry identified CE-induced refinement of capillary pores (<100 nm) with 18–22% increased bound water content, mercury intrusion porosimetry (MIP) and SEM studies highlighted macropore (>1 μm) coarsening [67]. This duality explains the strength reductions (12–18%) observed despite improved cement hydration.

Wetzel et al. established that matrix porosity gradients control water redistribution during curing with optimal pore networks minimizing differential shrinkage stresses at substrate interfaces [68]. Notably, CEs prolong carbonation kinetics, enabling gradual pore refinement through late-stage CaCO_3_ precipitation, which is a secondary densification mechanism that reduces total porosity versus conventional mortars [12]. Jumate et al. found that the introduction of CEs led to a decrease in the volume of small pores and an increase in the volume of large pores from 3% for the simplest plaster mortars (PM) up to 41.4% for PM with 1.0% CE [67]. These findings underscore the need for a holistic pore structure design when employing CEs. While air-entrainment enhances workability and interfacial adhesion, excessive CE dosing (>0.5%) risks creating percolation pathways that compromise durability. Advanced characterization combining in situ hydration monitoring with 3D pore network modeling could unravel the dynamic interplay between CE chemistry and void evolution.

**Figure 15:**
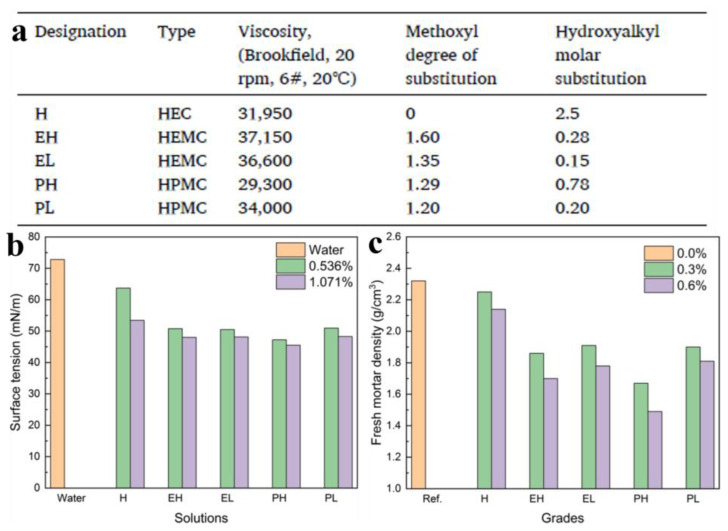
Basic parameters of tested CEs (**a**), effects of CEs grades on surface tension of their solutions (**b**), and fresh mortar density (**c**). Reprinted with permission from ref. [70]. Copyright (2021), Construction and Building Materials.

Zhang et al. analyzed the porosity and pore size distribution using MIP, and the MIP measurement showed that HEMCs increased the amount of micron-level pores and the porosity [69]. They also tested the change in mechanical properties of CSA cement mortars after the addition of CE. The results demonstrated that HEMCs improved the fresh state properties and tensile bond strength of both types of CSA cement mortars. However, the compressive strength of CSA cement mortars decreased greatly by the addition of HEMCs, and the flexural strength decreased slightly. Li and his co-workers systematically investigated the structure–property relationships of CEs in CSA cement mortars, revealing critical correlations between substituent parameters and mechanical performance [70]. In their findings, CEs (HEC and HPMC) have the least and most negative impact on these properties, respectively, which was closely associated with the MS of hydroxypropyl. Flexural strength development across curing stages showed strong nonlinear correlations with fresh mortar density, which inversely depends on the surface tension reduction in CE solutions. Special CEs exhibit surfactant-like characteristics due to the combination of both hydrophilic hydroxyl groups and hydrophobic alkyl substituents. Experimental results confirmed that CE incorporation reduces aqueous solution surface tension by up to 35.1%, with the magnitude dependent on both polymer type and concentration (Figure 15). This surface activity facilitates microbubble entrainment during mixing, significantly altering mortar density. Notably, high-substitution HPMC formulations reduced fresh mortar density by 28.0% compared to the reference samples, highlighting the dosage-dependent air-entraining capacity of CEs (Figure 15c). Substituent architecture governs performance outcomes: hydroxyalkyl MS emerges as the dominant factor influencing mechanical properties in methoxyl-containing CEs, while the DS of methoxyl only becomes relevant at low hydroxyalkyl MS levels. These findings underscore the hierarchical control of substituent parameters: the hydrophobic/hydrophilic balance, dictated by MS/DS ratios, determines interfacial interactions, air-void formation, and, ultimately, hardened matrix integrity.

**Figure 16:**
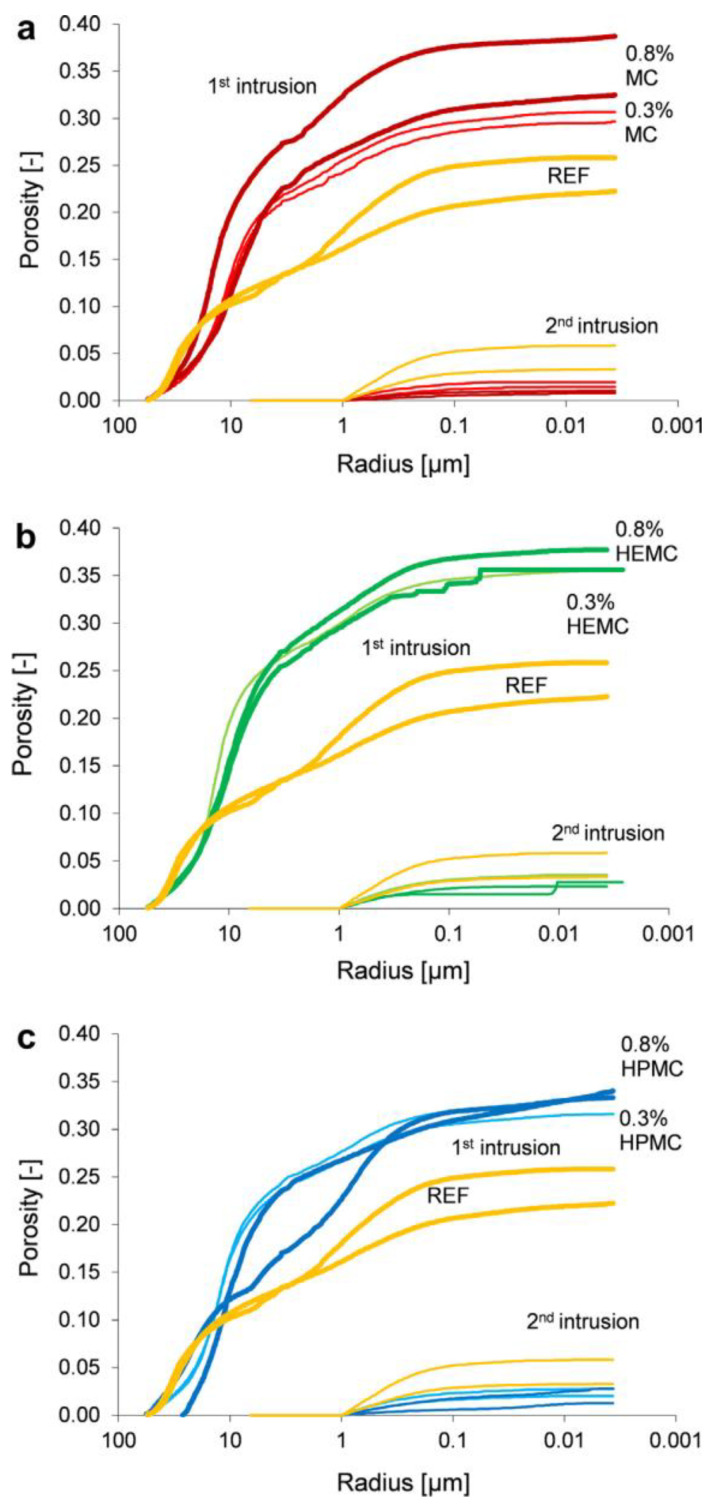
Equivalent pore size distributions for application on the substrate of concrete substrate with the addition of MC (**a**), HEMC (**b**) and HPMC (**c**). Reprinted with permission from ref. [72]. Copyright (2014), Cement and Concrete Composites.

Wang et al. employed calcium aluminate cement (CAC) as a model system to decouple the complex interactions between CEs and aluminate phases (e.g., C_3_A, C_12_A_7_) inherent in Portland cement [71]. Through a multi-technique approach combining IC, XRD, thermal analysis, and SEM, the study revealed that HEMC significantly alters CAC hydration kinetics. Although HEMC effectively prevented water loss during early hydration stages, microstructural analysis revealed a paradoxical effect, namely the formation of interconnected porous networks in hardened mortars. HEMC’s dual role as a water retention enhancer and hydration moderator arises from its competitive interactions with aluminate phases, stabilizing pore solutions while disrupting nucleation processes. The observed strength–porosity inverse relationship highlights the critical need for dosage optimization in CAC-based systems, particularly where early structural development is paramount. These findings provide a mechanistic framework for understanding CE effects in high-alumina cements, extending relevance to specialty applications requiring controlled setting behavior. Wyrzykowski et al. evaluated the effects of CEs’ addition on tile-adhesive mortars using MIP [72]. In their findings, the CEs’ addition predominantly increased total porosity through air entrainment (Figure 16), whereas the capillary and gel pore networks remained largely unaffected. Notably, substrate interaction critically influenced pore architecture. Application on high-water-absorption substrates triggered pore coarsening, creating interconnected channels that reduced mechanical interlock capacity. Paradoxically, tensile adhesion strength of tile-adhesive mortars increased gradually from 0.00 to 0.25–1.55, 0.06–1.53, and 0.05–1.37 N/mm^2^ with the addition of 0.3 or 0.8% MC, HEMC, and HPMC, respectively. The tensile adhesion strength decreased as the open time extended from 5 to 30 min, suggesting that interfacial chemistry modifications outweigh porosity-related weaknesses.

Brumaud et al. revealed that CE adsorption on cement grains within initial mixing stages creates steric barriers, delaying calcium silicate nucleation [73]. This interfacial interference reduces early yield stress development but enhances long-term hydration homogeneity through controlled water distribution. It is noted that the addition of a proper amount of CE is beneficial to enhance the properties of concrete and mortars, while too much CE might overly delay hydration or introduce excessive air, affecting and the structure and reducing density and strength.

Xu et al. have studied the effects of HMC on the flexural and compressive strength of cement mortar [74]. In their findings, the 1 d flexural strength and compressive strength of cement mortar increased from 0.353 and 1.36 MPa to 0.41 and 1.86 MPa by the addition of 0.1% HMC, and the 7 d compressive strength increased from 22.95 MPa to 24.53 MPa. With the increase in HMC concentration (0.2%), the 7 d flexural strength increased significantly from 6.26 MPa to 7.15 MPa. So, the addition of HMC could increase the early-stage mechanical properties of mortar. However, the flexural strength and compressive strength of cement mortars decreased with higher concentration (≥0.3%) of HMC or HEMC [75]. The 28 d flexural strength and compressive strength with all concentrations HMC or HEMC all decreased compared with mortars without cellulose ethers. Moreover, Wang et al. also studied the effects of HEMC on the 90 d, 180 d, and 360 d flexural and compressive strengths of cement mortars. They found that the flexural and compressive strengths decreased from about 9.5–10.5 MPa and 52.2–57.3 MPa to about 6.0–9.5 MPa and 12.1–35.7 MPa, indicating negative influence on the late flexural and compressive strengths due to the increase in porosity by the air-entraining role of CEs [76]. They also studied the effect of HEMC on the tensile bond strength. With the addition of 0.1% HEMC, the 3 d and 28 d tensile bond strengths increased significantly from about 0.40 and 0.43 MPa to 0.81 and 1.05 MPa, respectively. Moreover, the 90 d, 180 d, and 360 d tensile bond strengths also increased from about 0.75–9.2 MPa to 1.18–1.67 MPa, indicating positive effect on all the stages of tensile bond strength for mortars. Similarly, the 28 d tensile bond strength of cement mortars increased from about 0.42 MPa to 1.39 MPa by the introduction of HEMC, which was further increased to 1.49 by controlling air content [77].

More recently, CMC was found to have an obvious negative effect on the flexural strength and compressive concrete, which decreased, respectively, to 15% and 12% with the addition of 0.25% CMC [78]. For example, the increase in CE content from 0 to 1.5% reduced the 28 d compressive strength of ultra-high-performance concrete (UHPC) from 115 to 97 MPa, possibly due to the reduction in cement hydration, which was a result of the utilization of high superplasticizer content caused by competitive adsorption [79]. The addition of CE improved the adhesive strengths of the high strength cementitious grout for UHPC, and almost had no negative effect on the compressive and tensile strengths by the combination of defoamers [80]. The 3 d and 28 d compressive strength of concrete decreased by 11.5% and 3.1% with the introduction of HPMC due to the increase in hydration products caused by HPMC [81].

### 3.2. Other Applications and Effects of CEs

CEs are important materials with numerous applications in the pharmaceutical industry because of their extraordinary advantages, including high stability, biocompatibility, ease of solubility, and low toxicity [82]. Their safety is reinforced by negligible gastrointestinal permeability and a widespread use in FDA-approved formulations, where they function as stabilizers, viscosity modifiers, granulation binders, and tablet film-forming agents. Despite these advantages, CEs face challenges in pharmaceutical utilization due to poor aqueous dispersibility caused by rapid hydration and surface adhesiveness. Recent advancements focus on co-processing strategies to enhance dispersibility, with studies elucidating molecular mechanisms and technological interventions to address these limitations [83].

**Figure 17:**
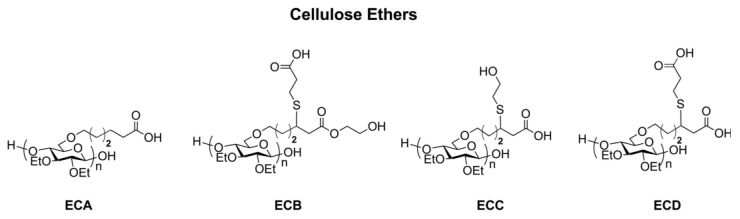
Molecular structures of synthesized cellulose ethers for the stabilization of drug solutions. Reprinted with permission from ref. [84]. Copyright (2018), Biomacromolecules.

CEs, with carboxylic acids substituents and side chains (Figure 17), could effectively delay drug crystallization from the supersaturated solutions and stabilize supersaturated drug solutions by affecting crystal nucleation and growth [84]. CEs have been used to prepare interpolymer complexes for the application of drug-delivery, which has been recently reviewed systematically by Keldibekova et al. [13]. Moreover, CEs were also used to fabricate novel nanofibers for the applications of drug delivery or scaffolds in tissue engineering [85]. HPMC is a typical CE utilized widely in drug formulations, especially for ocular and ophthalmic formulations. HPMC has been utilized as a viscosity enhancer agent in eye drops, gelling agent in injections, and matrix in different material forms, which have been systematically reviewed [86]. Three-dimensional printing is an attractive technology that could be used to prepare designed and complex hydrogels or scaffolds with living cells with extreme precision. Interestingly, CEs are amazing resources for 3D printing [87], and some CEs (e.g., MC, CMC, HPMC, and HEC) have been successfully 3D-printed into hydrogels or scaffolds for bone repair, which have been systematically reviewed [88,89,90,91].

CEs seem to resist complete desorption from the oil–water interface by the bile salts, which may make it difficult for the access of lipase to the interface. The interfacial characteristics play a key role on the control of digestion of emulsified lipids [92]. The types of CEs could affect the formation of crystal network and the textural and rheological properties of the emulsions [93]. It is noted that the original compact structure of the anhydrous milk fat (AMF) was softened by the presence of all cellulose types in direct spreadable food or in applications that require plastic properties. The emulsions could be used as a direct spreadable food or in applications that require plastic properties.

CEs also demonstrate a unique ability to resist bile salt-induced desorption at the oil-water interface, potentially hindering lipase accessibility and thereby modulating lipid digestion kinetics in emulsified systems. This regulatory effect is closely tied to their interfacial behavior, which critically governs enzymatic hydrolysis efficiency [92]. Furthermore, the CE type significantly influences emulsion microstructure, particularly the formation of crystalline networks that dictate texture and flow behavior. For instance, in anhydrous milk fat (AMF)-based systems, all tested cellulose variants disrupted the native compact architecture of AMF, enhancing plasticity and spreadability [93]. These modified emulsions show promise for applications requiring tailored rheological properties, such as ready-to-use spreadable products or formulations demanding controlled mechanical responses. The interplay between CE molecular structures and emulsion functionality underscores their versatility as multifunctional additives in lipid-based food matrices.

**Figure 18:**
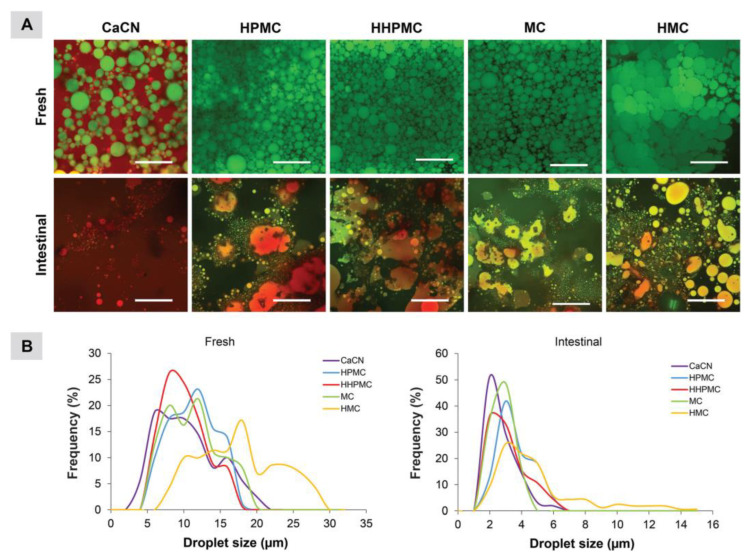
(**A**) Confocal micrographs of emulsions stabilized by different emulsifiers before (fresh emulsion) and after intestinal digestion phase. The scale bars are 60 μm. (**B**) Droplet size distribution of emulsions before (fresh) and after intestinal digestion phase. CaCN: calcium caseinate emulsion. Reprinted with permission from ref. [94]. Copyright (2017), Food & Function.

Borreani et al. investigated the role of CEs in stabilizing oil-in-water (O/W) emulsions, emphasizing their dual contributions to physical/oxidative stability and lipid digestion modulation [94]. The study revealed that CEs form a protective interfacial layer that segregates lipid substrates from pro-oxidants while thickening the aqueous phase to impede oxidant diffusion, thereby enhancing both physical integrity and oxidative resistance during storage. Notably, high-methoxyl methylcellulose (HMC) generated emulsions with exceptional structural consistency, maintaining rigidity during gastric digestion (Figure 18). Furthermore, HMC-modified emulsions exhibited slower lipid digestion kinetics compared to protein-stabilized counterparts due to the steric hindrance network formed by HMC at the oil-water interface, and delayed bile salt-induced displacement of CE molecules, which preserves the interfacial barrier. Such properties highlight HMC’s potential in weight management strategies by decelerating nutrient absorption and prolonging satiation. These findings align with broader studies on emulsion design, where interfacial engineering and aqueous phase viscosity are critical for modulating digestion profiles. The work underscores the versatility of CEs in tailoring emulsion functionality for both stability enhancement and targeted physiological outcomes.

CEs, such as CMC and HEC, are widely utilized in critical petroleum operations, including drilling, fracturing, enhanced oil recovery (EOR), and well completion [95,96,97]. In drilling fluids, CMC functions by thickening the fluid, suspending drill cuttings, and reducing fluid loss to stabilize wellbore walls, while its derivatives (e.g., HPCMC) exhibit salt resistance (adaptable to seawater or saturated brine) and high-temperature tolerance (up to 160 °C). During hydraulic fracturing operations, HEC significantly enhances fracturing fluid viscosity to transport proppants effectively, with its low-residue properties minimizing reservoir damage. In EOR, CMC improves sweep efficiency by adjusting the viscosity of displacing fluids, and its resistance to salinity and calcium ions makes it suitable for high-salinity reservoirs. Additionally, CEs serve as stabilizers in completion and cementing fluids, optimizing cement slurry performance to ensure wellbore integrity. Their comprehensive properties, such as rheological control and environmental adaptability, establish CEs as indispensable functional additives in petroleum extraction.

CEs have also been used in paint systems as thickeners due to their easy dissolution properties, wide availability, and excellent performance on viscosity regulation [98]. CEs have also been widely used as personal care and household products, including thickeners in shampoos, lotions, and toothpaste, as well as anti-redeposition agents in laundry powders and liquid detergents. Moreover, CEs can also be used to fabricate functional materials like films, fibers, hydrogels, and microspheres, among others. [9]. The related work has been studied in detail and systematically reviewed in recent years [99,100,101].

## 4. Summary and Prospect

CEs, as versatile, functional, and naturally derived polymers, have emerged as indispensable additives across industries, particularly in cementitious materials such as concrete and mortars. This review consolidates the synthesis strategies of CEs, including heterogeneous and homogeneous etherification processes, and underscores the pivotal role of structural parameters (like DS, M_w_, and functional groups) in dictating their properties. The heterogeneous method, though widely adopted for industrial-scale production, faces challenges in achieving uniform substitution, whereas homogeneous processes enable precise control over molecular architecture at the expense of scalability. CEs serve as multifunctional additives through enhancing water retention, rheological properties, and cohesion through mechanisms, including hydrogen bonding interaction, air-entrainment, pore structure modulation, and steric hindrance, among others. Their ability to balance workability and mechanical strength has revolutionized modern mortar formulations from self-leveling floors to high-adhesion tile adhesives. Beyond construction, CEs demonstrate promising applications in the biomedical and food sectors, leveraging their low toxicity and biocompatibility. However, persistent challenges hinder their full potential. These include (1) industrialization hurdles in homogeneous processes due to scalability and cost limitations; (2) difficulties in precise molecular design of specialized CEs; (3) overdosage-induced strength reduction in concrete/mortars; (4) incompatibility with advanced admixtures like polycarboxylate superplasticizers; and (5) environmental concerns associated with traditional synthesis methods. With the development of science and technology as well as the deeper cross-disciplinary integration, these issues will be solved through different pathways, such as green synthesis using eco-friendly etherification agents and process optimization, smart material design driven by AI, and in-depth collaborative efforts. The evolution of CEs hinges on transcending traditional synthesis-application paradigms. By further adopting green chemistry, precision design, and cross-sectoral synergies, CEs will continue to revolutionize industries, epitomizing the convergence of functionality, sustainability, and innovation. With continuous improvement and structural optimization, CEs will possibly be utilized in the fields of high-end paint or coating, flexible electronics, and aerospace, among other industries.

## Figures and Tables

**Table 1:** Types of CEs and their functional groups, application fields, and abbreviations.

Types	Specific Functional Groups	Major Application Fields	Abbreviations
Methyl cellulose	–OH or –OCH_3_	Food industry, biomedical, cosmetics	MC
Carboxymethyl cellulose	–OH or –OCH_2_COOH	Building, food or paper industry, biomedical, water treatment, textile	CMC
Ethyl cellulose	–OH or –OCH_2_CH_3_	Food industry, paper industry, biomedical, textile	EC
Hydroxy-ethyl cellulose	–OH or –OCH_2_CH_2_OH	Building, cosmetic, cleaning solutions, textile	HEC
Hydroxy-propyl cellulose	–OH or –OCH_2_CH(OH)CH_3_	Building, food or paper industry, biomedical, water treatment, textile	HPC
Cyanoethyl cellulose	–OH or –CH_2_-CH_2_-CN	Coatings, biomedical, paper industry	CEC
Ethyl hydroxyethyl cellulose	–OH or –CH_2_-CH_3_ or –CH_2_-CH_2_-OH	Biomedical, coatings	EHEC
Hydroxyethyl methyl cellulose	–OH or –CH_2_-CH_2_-OH or –CH_3_	Building, cosmetics, food or paper industry	HEMC
Hydroxypropyl carboxymethyl cellulose	–OH or –CH_2_-CHOH-CH_3_ or –CH_2_-COOH	Building, food industry, biomedical, cosmetics	HPCMC
Hydroxyethyl carboxymethyl cellulose	–OH or –CH_2_-CH_2_-OH or –CH_2_-COOH	Building, biomedical, food industry, cosmetics	ECMC

## Data Availability

No new data were created or analyzed in this study. Data sharing is not applicable to this article.
